# Design-based research for the development of a flexible learning environment

**DOI:** 10.4102/hsag.v24i0.1050

**Published:** 2019-09-30

**Authors:** Belinda van der Merwe

**Affiliations:** 1 Department of Clinical Sciences, Central University of Technology, Bloemfontein, South Africa

**Keywords:** design-based research, flexible learning, authentic learning and assessment, real life, mobile phone, effective performers

## Abstract

**Background:**

Students enrolled for the Bachelor of Radiography degree at the Central University of Technology Free State in Bloemfontein, South Africa, spent many weeks off campus at hospitals during workplace learning. A design-based research approach was applied to analyse an educational problem, namely that students apparently fail to apply the theory of radiation safety regulations to protect themselves and patients.

**Aims:**

The purpose of the study was to develop authentic activities and assessment for radiation safety requirements, to provide flexible learning in a blended environment to students off campus.

**Setting:**

The second-year radiography students of 2016 and 2017 responded off campus to the QuestionPro survey.

**Method:**

Authentic learning and assessment opportunities were contextualised and compiled to align with learning outcomes of the safety regulations as confirmed during a Delphi process. Flexible learning opportunities were delivered via electronic mail (email) and WhatsApp. Assessment responses from the students were received via mobile phones with a Web-based software tool, QuestionPro.

**Results:**

Of the 117 students, 92.3% responded to the QuestionPro survey. From the incorrect responses, learning areas that needed revision were identified, as well as the areas in the X-ray departments of the hospitals showing non-compliance with the requirements for radiation safety.

**Conclusion:**

The three outcomes of this design-based research included the formulation of design principles, a designed product in the form of a Website and professional development of the researcher. The design-based research principles that were tested involved the use of knowledge in real life and measuring if students were effective performers with acquired knowledge.

## Introduction

### The problem of applying theory in practice

The radiographer must be acquainted with the responsibilities of radiation workers proposed by the South African Department of Health, Directorate Radiation Control (DRC), in two documents, namely the ‘Code of practice for users of medical X-ray equipment’ (DRC 2015), plus ‘Requirements for licence holders with respect to quality control tests for diagnostic X-ray imaging systems’ (DRC 2012). The regulations guide the operators of X-ray equipment to protect staff and the patient to limit ionising radiation dose in order to adhere to the as low as reasonably achievable (ALARA) principle. The regulations decree that radiation workers must be educated in the safety and risks of ionising radiation. First-year radiography students are considered radiation workers from day 1, despite merely entering the profession. The final-year radiography students are potential licence holders of X-ray equipment.

A shortcoming was identified in the standardised education of radiography students at the Central University of Technology (CUT) as radiation workers. Therefore, the content and assessment of a course for radiation safety regulations were investigated and determined by a panel of experts in the field using a Delphi process (Van der Merwe 2015). The course was divided into learning units and included in the new bachelor’s degree at the CUT, with the first roll-out of the first year in 2014. The learning units were divided to spiral in depth and difficulty over the 4 years of the degree. First-year students are required to prove mastery of the theory by completing the written assessment and passing with a minimum mark of 80%. The students have the opportunity to repeat the assessments to achieve the set standard. Thus far, all students have successfully completed the written assessment.

However, it was alarming to note that first-year students completed radiation safety assessment on campus successfully but seemed to overlook that the requirements should be applied in the clinical setting once they were placed in hospitals as second-year students. The clinical tutors observed that the students did not necessarily apply the knowledge pertaining to the radiation regulations in the clinical setting. The reasons for the inability to apply the knowledge could be because of (1) the absence of authentic learning and assessment tasks regarding radiation safety requirements; or (2) insufficient monitoring because students work under supervision of qualified radiographers, who are not necessarily role models with regard to the application of rules and regulations. A need was identified to engage students with the content not only to reference the information, but also to apply the knowledge in real life.

When radiography students are not on campus, flexible technology-based learning opportunities need to be provided so that they can reflect on the huge amount of theory applied in the different placements in the imaging department. Authentic learning tasks can be employed as an organising component of the safety requirements module and can motivate students to learn (Herrington, Reeves & Oliver 2010).

The learning approach of this project was based on theories of situated learning and authentic learning. Situated learning is a general theory of knowledge acquisition, which means that a student’s learning takes place during social interaction by a process of increased participation and collaboration (Lave & Wenger 1990). Under supervision of a professional in the imaging department, the radiography student may be guided to master practical requirements and apply theory in practice. The elements of authentic learning that were considered to address the educational problem included the provision of authentic tasks and activities, supporting collaborative construction of knowledge, and providing authentic assessment of learning within the tasks (Herrington 2006).

The purpose of the study was to develop authentic activities and assessment for radiation safety requirements, in order to provide flexible learning in a blended environment to students off campus. Blended learning has been defined as ‘the thoughtful integration of classroom face-to-face experiences with technology-enhanced learning experiences’ (Van der Merwe et al. 2015:11).

For meaningful learning to take place, the pedagogical focus requires that learning tasks must be aligned with the learning outcomes and teaching activities. The students’ prior knowledge and competencies must be considered, as well as the technologies available to the group of students (Bozalek & Ng’ambi 2015). It was imperative to make decisions regarding the software that would be suitable to engage the students so that meaningful learning would take place and all students could be involved. The idea was to provide a learning environment website for radiation safety requirements to give students access to content regarding the requirements while placed in the hospitals for clinical practice. The strategy was further to complement the website with authentic tasks accessible via mobile phone, enabling students to engage with the content in order to master the theory of radiation safety requirements through these tasks.

The design-based approach that was followed to execute the research is explained below. The results are reported in a manner that the various cycles typical of design-based research are depicted.

#### Design-based research approach

Design-based research has its focus on real-world problems, with the overall goal of improving learning, rather than proving that one pedagogical approach is more effective than another. In the first phase of design-based research, a problem is analysed in depth in consultation with the practitioners or teachers involved. A solution is then designed according to theoretical principles and with knowledge of recent technological availability. The proposed solution (also referred to intervention) is then implemented in two or more repetitions, with adjustments and improvements made between implementations, so that the emphasis remains on finding the best way to present the subject in the particular pedagogical context. The last phase is the creation of design principles based on the knowledge gained from the theory and practice of and reflection on the previous phases (Herrington et al. 2008). Therefore, design-based research has three potential outcomes, namely (1) design principles, (2) designed products: the physical representations of the learning environment and (3) societal outputs, such as professional development and learning. The design-based research approach as described by Brown (1992) and Collins (1992) has been summarised by Reeves (2006) into four phases, namely to analyse a problem in collaboration with practitioners, to develop solutions informed by draft design principles and to implement the solutions in practice in different cycles. The fourth phase reflects on the implementation of the solution to confirm a design principle.

#### Analysis of a practical educational problem

The first phase of designed-based research is to focus on the analysis of a significant educational problem. In this study, the problem was pinpointed that radiography students spend weeks off campus at hospitals during workplace learning, seemingly challenged to apply the theory of radiation safety to protect themselves as well as the patients against radiation.

#### Collaboration by means of a Delphi survey

As part of a larger study, lecturers and experts in the field of radiography were involved to explore the vital content for students to apply and implement radiation safety requirements in practice (Van der Merwe 2015). The first step was to determine the content of the requirements relevant for the South African context. The content was aimed at two different categories, namely basic for entry radiation workers, and advanced for potential licence holders of X-ray equipment.

In addition to diagnostic radiographers who are specialists in the field as clinical practitioners or lecturers, a physicist assisted with the decision pertaining to the relevance of content applicable to radiation safety requirements for students. The experts involved provided confirmation of the conceptualisation of the content of the training and assessment. The opinion of the experts reached consensus regarding the content of the radiation safety regulations training course by means of a Delphi technique.

Skulmoski, Hartman and Krahn (2007) indicated that because of the flexibility of the Delphi technique, the method can be applied in different disciplines. These authors described three-round studies with successful outcomes (Skulmoski et al. 2007), but the current study, however, involved four rounds before consensus was reached and stability declared. Stability can be declared when the panellists do not change their opinion in two rounds (Linstone & Turoff 1979). In the last round, the panellists were informed that they had the opportunity to alter their choice, but nobody indicated any changes.

The Delphi process involved a quantitative approach to determine the learning outcomes for a radiation safety training course for diagnostic radiography students. A section of the Delphi survey was focused to determine the opinion of the panellists regarding the presentation and assessment strategies of the training course. The criteria statements of the survey involved the opinion of the panellists on whether the learning and teaching activities should be presented as lectures in a classroom setting, online or as a combination of the two modalities. The panellists strongly agreed that all the criteria on which consensus was reached in the survey had to be included and confirmed by assessment (Van der Merwe, Kruger & Nel 2017).

The panel could not reach consensus on the delivery of the basic assessment in electronic format. A comment was that one part of the assessment could be performed via e-learning, but a clinical component should be included, such as an objective-structured clinical examination (OSCE). The panel agreed that the advanced assessment could be delivered in electronic format. The panellists confirmed that the Delphi criteria statements included the requirements to safely operate diagnostic X-ray equipment in clinical practice, and to equip students to comply with regulations as future professionals. The valuable guidance of the Delphi panellists reiterated that neither distance learning nor self-study should be considered when students need hands-on training. Specific comments confirmed the importance to record evidence of mastery of content by means of assessment. The importance of distributing the workload between the years of study and repetition to provide students with the opportunity to reach a high level of understanding was emphasised (Van der Merwe et al. 2017).

The insightful comments from the panellists resulted in a final list of criteria for radiation safety requirements that were divided into basic and advanced training and assessment. Although the comments regarding the understanding of the content were considered in the planning of the teaching and learning activities, it was observed that the students were not able to apply all the theory in practice. This necessitated that authentic learning activity ideas had to be explored. The following questions needed to be addressed: *What software will be suitable to engage all the radiography students in practice? Will meaningful learning take place?*

### The literature review to find solutions

A literature review focused on authentic learning and assessment, mobile learning software currently available, teaching strategies and design principles to develop the learning environment. The EBSCO host database, Medline database, EMBASE, SACat, Academic Search Premier, Science Direct and Best Evidence medical database were consulted to identify relevant articles.

Flexible learning for this article was explored because increased movement and flexibility influence how students use technology to support their learning. Students value the fact that there are no longer restrictions to be in a computer laboratory or on campus, and having the option to work on assignments off campus by accessing the Internet and online resources on their own devices (Brown & Pallitt 2015). The classroom activities are thus extended outside the classroom to provide a flexible learning environment.

#### Authentic learning and assessment

The *Guide to authentic e-learning* by Herrington et al. (2010) was a valuable resource in the design of the mobile content. Authentic learning implies that ‘learners must be engaged in an inventive and realistic task that provides opportunities for complex collaborative activities’ (Herrington et al. 2010:1). It is a misconception that authentic learning can only take place in a real work setting, as it is also suitable for computer- and Web-based delivery. The technologies must be used as cognitive tools for learning rather than substitute delivery platforms (Herrington et al. 2010).

The researcher realised that to provide a mobile-friendly learning environment, a website for radiation safety requirements, for example, could give radiography students access to content regarding requirements during placement in hospitals for clinical practice. However, the availability did not mean that they would engage with the content. It was important to confirm if authentic tasks accessible via mobile phone could motivate students to apply theory in practice. The student must therefore be enabled to engage with the content in order to master the theory of radiation safety requirements through these tasks.

Herrington et al. (2010) reiterated that in the designing of e-learning courses, it is not sufficient to only provide examples from real-world situations to illustrate concepts, but the purpose of and motivation for learning must be provided. The lecturer must ask questions pertaining to where and how the knowledge will be used to plan the activities. Students must have different perspectives from various points of view. The responses that students must submit have to involve reflection on their own practice, and the implementation and application of theory should be judged by the answers.

The authentic assessment that was considered for the students required that students are effective performers with the acquired knowledge. Authentic assessment is valid when the test simulates real-world examinations of ability. The nine elements of authentic learning (Herrington 2006) that can guide the planning of flexible learning are:

- it provides authentic contexts that reflect the way the knowledge will be used in real life
- it provides authentic tasks and activities
- it provides access to expert performances and the modelling of processes
- it provides multiple roles and perspectives
- it supports collaborative construction of knowledge
- it promotes reflection to enable abstractions to be formed
- it promotes articulation to enable tacit knowledge to be made explicit
- it provides coaching and scaffolding by the teacher at critical times
- it provides for authentic assessment of learning within the tasks.

The assessment tasks for the radiation safety requirements were planned by considering these elements to constantly reflect on how to force the student to apply theory in day-to-day activities in the clinical setting.

#### Software for mobile content on demand: The flexible learning environment

Mobile technologies, such as mobile phones, are technologies that most students have in their pocket, an observation that has been supported by statistics that mobile penetration in South Africa is around 128%. Over 80% users use their smartphones to access the Internet (Thomas 2014). The Adobe PDF Pro IX software was purchased in 2015 to create fillable forms in PDF format. The first learning activity was compiled by employing this software. However, it was very time-consuming and to create a fillable form was challenging. The instructional designer then introduced the QuestionPro (2016) software. In the pilot phase that involved supplying the learning tasks to the students, QuestionPro, a Web-based software tool, was used. This software enabled students to have access to survey response links via email, SMS or WhatsApp.

Online surveys and polls can be created with QuestionPro. Responses can be collected even offline using the Android mobile survey application. Results can be analysed with a full set of reporting features such as a real-time summary, pivot tables, segmentation tools, trend analysis and text analytics. The survey data can be exported directly to Microsoft (MS) Excel, SPSS or comma separated values (CSV). The results can be shared by means of formatted MS Word and MSPowerPoint reports, or by generating information graphics (QuestionPro 2016).

#### Teaching strategies on the digital platform

As more free academic content is accessible to students, the content delivery is of less importance to students than the support of the lecturer (Bates 2014). The lecturer must first understand what his or her own epistemological perspective of learning is, because that is the driver of teaching. The current study used constructivism and connectivism. Constructivists believe that in order for a student to understand, the student must be able to relate new information to existing knowledge and furthermore integrate it. Connectivism is the combined links in a network to obtain new knowledge. The knowledge is further generated outside the level of individual participants, and is continually changing (Bates 2014).

Even if we teach academic content that is applied in a practical context, the support from the lecturer with learning must consider the importance to develop the responsibility of students. This responsibility includes learning by themselves, acquiring new skills and applying theory as professionals. This is necessary because information overload makes it impossible to memorise all the data, and students need to be able to stay abreast of development as professionals (Bates 2014).

In the current digital environment, the lecturer can make the mistake to think that when digital teaching methods are selected, for example video or scheduling students in the workplace, learning will take place. However, this will not necessarily guarantee understanding or learning, and therefore a combination of methods should be executed (Bates 2014). The author further provides ideas for learning with mobile devices, namely creating multimedia projects or submitting e-portfolios as a form of assessment.

The movement is away from teaching models to design models. During the design, a number of factors must be taken into consideration and it should be kept in mind that students learn better in the digital environment under the following conditions:

- when pictures accompany words
- when the inclusion of words, sounds and pictures is rather less than more
- when words and pictures that match are displayed close together on the screen
- when important material is organised in a way that the student can easily follow
- when a multimedia learning unit (video) is divided into shorter sections rather than a lengthy lesson
- when the main principle is lectured before the detail of application is provided in a multimedia learning unit.

Bates (2014) recommended to avoid overload, and to focus on what students need to do to engage with the content to promote their ability to apply the content. It is further important to plan for activities with some form of feedback.

#### Design principles to develop the learning environment

The research question was focused on whether it would be possible to deliver authentic learning and assessment tasks on radiography students’ mobile devices so that meaningful learning would take place. Herrington (2006) provided examples of authentic learning, for example (1) to provide authentic tasks and activities and (2) to provide multiple roles and perspectives to reflect how knowledge can be used in real life. Wiggins (1990) characterised authentic assessment as a task that will require students to be effective performers with acquired knowledge, presents the student with a full array of tasks and involves well-structured challenges that help students rehearse for the complex ambiguities of professional life.

The design principles can include real-world application in mobile learning environments where students are placed in clinical practice. It therefore means that students are mobile and through their own mobile phones, knowledge is produced (Herrington, Herrington & Mantei 2009). Draft principles suitable for this study were selected from the literature, as outlined in Table 1.

**TABLE 1:** Draft design principles for the study.

Principle	Reference
**Radiation may best be facilitated by learning environments that are:**	
Draft Principle 1: Flexible and blended (website accessible via a mobile phone)	Van der Merwe et al. 2015
Draft Principle 2: Authentic task reflects the way the knowledge will be used in real life (provide authentic tasks and activities in fillable PDF format or delivered by QuestionPro)	Herrington 2006
Draft Principle 3: Promote reflection to enable abstractions to be formed	Herrington et al. 2010
Draft Principle 4: Situated learning via collaboration (supervision and peer collaboration)	Lave & Wenger 1990
Draft Principle 5: Tasks from different perspectives with different solutions. Different methods must be combined (e-portfolio)	Bates 2014
Draft Principle 6: Complex tasks over a period of time	Herrington et al. 2010

*Source*: Adapted from Herrington, J., 2006, Design-based research methods. Links and resources, viewed 14 November 2017, from http://authenticlearning.info/DesignBasedResearch/Design-based_research.html, template: http://authenticlearning.info/DesignBasedResearch/Design-based_research_files/DBRTemplateFull_1.docx.

## Methods

### Method of the intervention solution in practice

The second-year students of 2016 (*n* = 57) and 2017 (*n* = 60) were included in the study. Permission to distribute the questionnaires to the student population at the institution was obtained. The students’ consent to participate was informed and voluntary. Design-based research is not a methodology, but a research approach. While both qualitative and quantitative methods may be used, it is worth noting that ‘[*d*]esign researchers do not emphasise isolated variables. While design researchers do focus on specific objects and processes in specific contexts, they try to study those as integral and meaningful phenomena’ (Van den Akker et al. 2006:5).

Learning tasks were communicated via the mobile environment and made accessible to all second-year students by providing PDF and MS Word documents via email and WhatsApp. The students (*n* = 117) then received a questionnaire survey via QuestionPro to respond. The questionnaire was only provided after a specified time so that students had time to accumulate information, collaborate and reflect on their own practices. The link to the survey software was provided via email and WhatsApp.

### Design of the learning environment

Four learning activities were planned for the second-year students to align with learning outcomes confirmed during the Delphi process. The design principles were used as a guide to formulate tasks so that students could explore the relevance of theory in practice. The tasks included the following:

- Radiation safety requirement task 1: Shielding.
- Radiation safety requirement task 2: Environment requirements.
- Radiation safety requirement task 3: Exposure index.
- Radiation safety requirement task 4: Licencing requirements.

**FIGURE 1:**
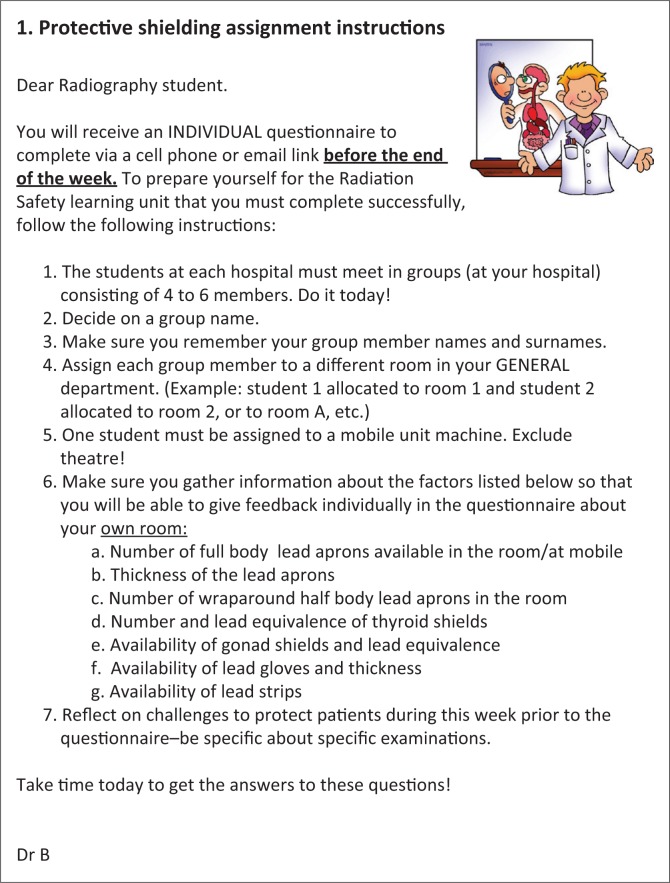
PDF instruction that the students received via email and WhatsApp.

The activity for shielding will be described as an example. The shielding activity was planned in order to guide the students to reflect on the lead aprons available in the relevant imaging departments. They had to discuss ideas to make sure that the radiation principle is implemented by using shielding to protect the patient. Figure 1 is an example of the PDF instruction that the students received. After the opportunity to engage in the activity, authentic assessment questions were peer-reviewed and formulated with the QuestionPro software to compliment the activity.

The QuestionPro survey consisted of 13 questions pertaining to identification of the student, the hospital where the students were placed, group name, the specific room to which the student was allocated, the number of lead aprons available, the thickness of the aprons and the application of theory of shielding in the X-ray room. The final question required the student to reflect on ideas to make sure the ALARA principle had been implemented by using shielding and to refer to their own strength and weakness in implementation of shielding for patients.

Once a learning environment or intervention has been designed and developed, the next phase of design-based research is the implementation and evaluation of the proposed solution in practice. Data collection took place by providing activity instructions and delivering the QuestionPro survey via email and as a WhatsApp message. Analysis of data for the first activity, for instance, included to find the availability of a thyroid shield in the department in the specific room to which the student was assigned for that week. The responses were recorded, and the results were automatically displayed with the QuestionPro software in graph format. The data could be exported via the software to MS Word or Excel to be analysed.

### Ethical considerations

Ethical approval to execute the research project was obtained from the ethics committee of Central University of Technology (ECUFS 74/2013).

## Results and discussion

Of the 117 second-year radiography students (*n* = 117), 108 (92.3%) responded to the QuestionPro survey. From the incorrect responses, the researcher could identify learning areas that needed revision. The survey also indicated areas in the hospitals that did not meet the requirements for safety, which was an additional benefit of the exercise. Although unplanned in the design phase, the students then became involved to find solutions for communicating the non-compliance and addressing the resource challenge in the departments. This deepened their engagement with the theory. Exhibit 1 represents an example of the students’ reflections regarding the room they were allocated to for the assignment.

The students were also engaged in the process to refine the activity and questionnaire. Eighty-eight (75.2%) of the students completed a satisfaction survey regarding the QuestionPro mobile questionnaire after they had completed the clinical block period. Of these students, 26% indicated that they made use of the survey link via mobile phone, 29% via email and 53% used both applications. With regard to specific aspects of the activity, 94% indicated that the instructions were clear, 80% that they enjoyed the exercise and 94% that they did make use of the opportunity to consult peers. The responses indicated that they needed more time for completion of the task, which will be adapted for the next student group. A valuable application of theory in practice that was indicated by 88% of the participants was the warm-up procedure of the X-ray machines.

The need for a reference source on demand was confirmed by responses from students regarding the radiation safety requirements. Therefore, the practical output of this design-based research was to convert the content to be suitable for a website (Radiation Safe Radiographers, available at URL http://radiationsafe.co.za/wp-login.php?redirect_to=http%3A%2F%2Fradiationsafe.co.za) accessible by means of a mobile phone. The collaboration integral to the process of designing and accomplishing a design-based research project had an additional benefit in that it enhanced the professional development of all people involved, not only the students. The researcher developed abilities and experience in the areas of website design and planning, and acquired skills to develop PDF forms for mobile delivery.

Once a learning environment or intervention has been implemented, evaluated and refined in cycles, the final phase is to produce design principles that can inform future development and implementation decisions. The draft principles (Table 1) that guided the researcher in the design of the flexible learning environment needed to be revisited. The six draft design principles were therefore confirmed as the design principles (Table 2) that will shape future authentic activities and assessment.

**TABLE 2:** Design principles reflected in practice.

Design principles	How each will be implemented in the learning environment
Principle 1: Flexible and blended (website accessible via a mobile phone)	A flexible mobile learning environment will be created that will allow students to have access to information on demand to confirm the correct application of theory.
Principle 2: Authentic task reflects the way the knowledge will be used in real life (provide authentic tasks and activities in mobile fillable format)	Knowledge will be used in real life and investigate the learning from different perspectives or departments in real life and measure if they are effective performers with acquired knowledge. The theory will be tested in practice – the student will be required to check the specific technical requirements in each department.
Principle 3: Promote reflection to enable abstractions to be formed	The responses that must be submitted will involve reflection on own practice and implementation and application of the theory. Each student will reflect on a different implementation, for example shielding, Exposure Index.
Principle 4: Situated learning via collaboration (supervision and peer collaboration)	Students will complete assessment tasks to be relevant to what they experience in the clinical setting and will be guided to reflect on practices in collaboration with peers. They will reflect on role models in practice with specific examples.
Principle 5: Tasks from different perspectives with different solutions. Different methods must be combined (e-portfolio)	Advanced students will have the opportunity to explore different ways to execute quality testing – different solutions will be accepted and encouraged. Submit proof in e-portfolio.
Principle 6: Complex tasks over a period of time	Requires students to be effective performers with acquired knowledge. Application of theory in practice will be recorded on a checklist. Submit proof of evidence portfolio.

*Source*: Adapted from Herrington, J., 2006, Design-based research methods. Links and resources, viewed 14 November 2017, from http://authenticlearning.info/DesignBasedResearch/Design-based_research.html, template: http://authenticlearning.info/DesignBasedResearch/Design-based_research_files/DBRTemplateFull_1.docx.

## Limitations

Over the 2 years, nine students did not complete the survey because of absence from work. During the next rounds, the student responses were checked against a class list. The process must be monitored to ensure that all students respond. The survey must be made available on mobile phone and via email. The satisfaction survey was not completed by all. Some students did submit twice because they were not sure if the survey submission was successful. This problem was addressed and improved in the next round.

## Conclusion

The design-based research focused on the real-world problem of application of safety regulation theory in clinical practice. A possible solution was designed and implemented as a website and tasks were delivered on the student’s cell phones while off campus. A flexible learning environment was thus designed and implemented with QuestionPro to engage the student to apply theory in practice. The last phase was the creation of design principles based on the knowledge gained from the theory and practice of and reflection on the previous phases.

A flexible, mobile learning environment was created with a design-based research approach that allowed students to have access to information on demand. Authentic activities and assessment were submitted via mobile phone to engage the student and promote learning in the clinical setting. The design-based principles that will be refined in future include creating more flexible mobile learning environments that will allow students to use knowledge in real life and measuring if students are effective performers with the acquired knowledge.

## References

[CIT0001] Bates A.W, 2014, *Teaching in a digital age. Guidelines for designing teaching and learning*, BCcampus Open Education, Vancouver, viewed 14 November 2017, from http://opentextbc.ca/teachinginadigitalage/.

[CIT0002] Bozalek V. & Ng’ambi D, 2015, ‘The context of learning with technology’, in Kilfoil W.R. (ed.), *Moving beyond the hype: A contextualised view of learning with technology in higher education*, pp. 3–6, Universities South Africa, Pretoria.

[CIT0003] Brown A.L, 1992, ‘Design experiments: Theoretical and methodological challenges in creating complex interventions’, *Journal of the Learning Sciences* 2(2), 141–178. 10.1207/s15327809jls0202_2

[CIT0004] Brown C. & Pallitt N, 2015, ‘Personal mobile devices and laptops as learning tools’, in Kilfoil W.R. (ed.), *Moving beyond the hype: A contextualised view of learning with technology in higher education*, pp. 25–28, Universities South Africa, Pretoria.

[CIT0005] Collins A, 1992, ‘Towards a design science of education’, in Scanlon E. & O’Shea T. (eds.), *New directions in educational technology*, pp. 15–22, Springer-Verlag, Berlin.

[CIT0006] Directorate Radiation Control (DRC), 2012, *Requirements for licence holders with respect to quality control tests for diagnostic X-ray imaging systems*, viewed 14 November 2017, from http://rssa.co.za/downloads/doc_download/1511-diagnostic-qc-modified-jan-2012-vers-7.

[CIT0007] Directorate Radiation Control (DRC), 2015, *Radiation control code of practice for users of medical X-ray equipment*, viewed 14 November 2017, from http://radiationsafe.co.za/wp-content/uploads/2017/08/1.3-Code-of-practice-for-users-of-medical-x-ray-equipment-2015.pdf.

[CIT0008] Herrington J, 2006, Design-based research methods. Links and resources, viewed 14 November 2017, from http://authenticlearning.info/DesignBasedResearch/Design-based_research.html, template: http://authenticlearning.info/DesignBasedResearch/Design-based_research_files/DBRTemplateFull_1.docx.

[CIT0009] Herrington A., Herrington J. & Mantei J, 2009, ‘Design principles for mobile learning’, in Herrington J., Herrington A., Mantei J., Olney I. & Ferry B. (eds.), *New technologies, new pedagogies: Mobile learning in higher education*, pp. 129–138, University of Wollongong, Wollongong, viewed 14 November 2017, from http://ro.uow.edu.au/edupapers/91/.

[CIT0010] Herrington J., Mantei J., Herrington A., Olney I.W. & Ferry B, 2008, *New technologies, new pedagogies: Mobile technologies and new ways of teaching and learning*, viewed 14 November 2017, from https://ro.uow.edu.au/cgi/viewcontent.cgi?article=1342&context=edupapers.

[CIT0011] Herrington J., Reeves T.C. & Oliver R, 2010, *A guide to authentic e-learning*, viewed 14 November 2017, from http://researchrepository.murdoch.edu.au/id/eprint/1903/.

[CIT0012] Lave J. & Wenger E, 1990, *Situated learning: Legitimate peripheral participation*, Cambridge University Press, Cambridge, UK.

[CIT0013] Linstone H.A. & Turoff M, 1979, *The Delphi method: Technique and application*, Addison-Wesley, London.

[CIT0014] QuestionPro, 2016, *How it works*, viewed 14 November 2017, from http://www.questionpro.com/.

[CIT0015] Reeves T.C, 2006, ‘Design research from a technology perspective’, in Van den Akker J., Gravemeijer K., McKenney S. & Nieveen N. (eds.), *Educational design research*, pp. 52–66, Routledge, London.

[CIT0016] Skulmoski G.J., Hartman F.T. & Krahn J, 2007, ‘The Delphi method for graduate research’, *Journal of Information Technology Education* 6, 1–21. 10.28945/199

[CIT0017] Thomas S, 2014, *16 graphs that shed new light on the South African smartphone space*, viewed 08 June 2016, from https://memeburn.com/2014/08/16-graphs-that-shed-new-light-on-the-south-african-smartphone-space/.

[CIT0018] Van den Akker J., Gravemeijer K., McKenney S. & Nieveen N, 2006, ‘Introducing educational design research’, in Van den Akker J., Gravemeijer K., McKenney S. & Nieveen N. (eds.), *Educational design research*, pp. 3–7, Routledge, London.

[CIT0019] Van der Merwe A., Bozalek V., Ivala E., Nagel L., Peté A. & Vanke C, 2015, ‘Blended learning with technology’, in Kilfoil W.R. (ed.), *Moving beyond the hype: A contextualised view of learning with technology in higher education*, pp. 11–15, Universities South Africa, Pretoria.

[CIT0020] Van der Merwe B, 2015, ‘Radiation safety regulations training and assessment for diagnostic radiographers in South Africa’, Unpublished PhD thesis, University of the Free State, Bloemfontein.

[CIT0021] Van der Merwe B., Kruger S.B. & Nel M.M, 2017, ‘Radiation safety requirements for training of users of diagnostic X-ray equipment in South Africa’, *African Journal of Health Professions Education* 9(3), 123–127. 10.7196/AJHPE.2017.v9i3.691

[CIT0022] Wiggins G, 1990, *The case for authentic assessment*, ERIC Clearinghouse on Tests, Measurement, and Evaluation, Washington, DC, viewed 14 November 2017, from https://eric.ed.gov/?id=ED328611.

